# Assessment of T1 and T2 Relaxation-Time Changes in NMIBC Tissue After 5-ALA Photodynamic Therapy Using Quantitative Magnetic Resonance Imaging

**DOI:** 10.3390/biomedicines13122867

**Published:** 2025-11-24

**Authors:** Dominik Godlewski, Klaudia Dynarowicz, Adrian Truszkiewicz, Michał Osuchowski, Tomasz Kubrak, Dorota Bartusik-Aebisher, Agnieszka Przygórzewska, Jakub Szpara, David Aebisher

**Affiliations:** 1 Medical Center in Łańcut, 37-100 Łańcut, Poland; remediumplus@o2.pl; 2 Department of Biochemistry and General Chemistry, Faculty of Medicine, Collegium Medicum, University of Rzeszów, 35-959 Rzeszów, Poland; kdynarowicz@ur.edu.pl (K.D.); tkubrak@ur.edu.pl (T.K.); dbartusikaebisher@ur.edu.pl (D.B.-A.); 3 Department of Photomedicine and Physical Chemistry, Faculty of Medicine, Collegium Medicum, University of Rzeszów, 35-959 Rzeszów, Poland; atruszkiewicz@ur.edu.pl; 4 Department of Pathomorphology, Faculty of Medicine, Collegium Medicum, University of Rzeszów, 35-959 Rzeszów, Poland; mosuchowski@ur.edu.pl; 5 English Division Science Club, Faculty of Medicine, Collegium Medicum, University of Rzeszów, 35-959 Rzeszów, Poland; ap117623@stud.ur.edu.pl (A.P.); js126214@stud.ur.edu.pl (J.S.)

**Keywords:** NMIBC, PDT, MRI, bladder, relaxation, mapping

## Abstract

**Background/Objectives**: Non-muscle-invasive bladder cancer (NMIBC) accounts for the majority of newly diagnosed bladder cancers and is characterized by a high recurrence rate despite standard treatments. Photodynamic therapy (PDT) using 5-aminolevulinic acid (5-ALA) offers a promising alternative; however, objective methods for monitoring therapeutic response are needed. Quantitative magnetic resonance imaging (MRI), particularly T1 and T2 relaxation mapping, may provide non-invasive biomarkers of tissue response to PDT. **Methods**: In this ex vivo study, 50 samples obtained during transurethral electro-resection of bladder tumors were analyzed using a 1.5 Tesla MRI scanner. Twenty-five healthy control tissues (n = 25) served as the control group. Twenty-five NMIBC tissues were analyzed twice: before and after 5-ALA-PDT. T1 and T2 relaxation times were measured, and regions of interest (ROIs) were manually segmented to obtain quantitative values. Statistical analyses were performed to assess the significance of differences observed between study groups. **Results**: T1 relaxation times significantly differed between groups: 1351.7 ± 271.1 ms in healthy tissue, 727.7 ± 145.0 ms in untreated NMIBC, and 368.9 ± 65.2 ms in NMIBC after PDT (*p* < 0.0001). T2 values were 93.5 ± 20.3 ms (healthy), 78.5 ± 20.4 ms (NMIBC), and 55.7 ± 6.1 ms (NMIBC_PDT), with significant reduction after PDT (*p* < 0.01). **Conclusions**: Quantitative T1 and T2 mapping via MRI is a sensitive and non-invasive method for detecting structural and biochemical changes in bladder tissue following 5-ALA-PDT. These relaxation times may serve as reliable biomarkers for therapeutic response, facilitating in real-time monitoring and personalized treatment planning for NMIBC. Further in vivo studies are warranted to validate these findings and integrate them into clinical practice.

## 1. Introduction

### 1.1. Epidemiology of NMIBC and Clinical Classification

Cancer, a large and heterogeneous group of malignancies, is becoming an increasingly important cause of premature mortality worldwide [1]. Bladder cancer is one of the most common urological cancers. Approximately 3.0% of all new cancer diagnoses and 2.1% of all cancer deaths are attributable to bladder cancer [2]. Histologically, bladder cancers include urothelial carcinoma, squamous cell carcinoma, adenocarcinoma, urachal carcinoma and small cell neuroendocrine carcinoma [3]. The most common bladder cancer, which accounts for more than 90% of all bladder cancers in the population of industrialized countries, is urothelial carcinoma of transitional epithelial origin [4]. Clinically, a distinction is made between non-invasive forms, confined to the mucosa or lamina propria, with a high risk of recurrence, and invasive forms (MIBC), infiltrating the bladder muscle, and carry a risk of metastasis and a much poorer prognosis [5]. NMIBC represents the majority of cases, accounting for ∼75% of newly diagnosed cases of bladder cancer [6]. Currently, transurethral resection of the bladder tumor (TURBT) with histopathological evaluation is considered the gold standard for the diagnosis of NMIBC, which allows precise staging and planning for possible re-do surgery in case of high risk of recurrence [7]. The classic histopathological division of NMIBC, based on the depth of infiltration (TNM system), distinguishes three basic categories of lesions: carcinoma in situ (Tis/CIS), pTa tumors and pT1 tumors. Carcinoma in situ is a multifocal, flat lesion with a high degree of malignancy confined exclusively to the epithelial layer of the mucosa, without forming papillae or infiltrating deeper layers of the bladder wall. Tumors of pTa are papillary, superficial tumors limited to the epithelium that do not cross the basement membrane and show no signs of invasion. In contrast, pT1 tumors are characterized by infiltration of the submucosa (lamina propria) but stop before the muscularis propria layer [8].

### 1.2. Modern Diagnostic Methods and Treatment

In the diagnosis of NMIBC, in addition to standard methods, several modern techniques are also employed, such as photodynamic diagnosis using 5-aminolevulinic acid (5-ALA) [9], analysis of *TERT* promoter mutations and oncogenic FGFR3 mutations in urinary cells using next-generation sequencing (NGS) [10], detection of *TERT* mutations in urine samples with the ddPCR platform [11], and molecular PET/CT imaging with novel radiopharmaceuticals, which enables a more accurate assessment of the metabolic activity of lesions invisible to conventional cystoscopy [12].

Treatment of NMIBC begins with TURBT, often followed by a second procedure (second-look TURBT) in high-risk cases, which improves the completeness of resection and reduces the risk of residual disease [13,14]. Intravesical administration of Bacillus Calmette-Guérin (BCG) remains the mainstay of adjuvant therapy, as it reduces the risk of recurrence and progression [15]. In cases of contraindications or in patients with low- or intermediate-risk disease, regimens with mitomycin C or other cyto-statics are used instead [16,17]. In patients who do not respond to BCG, particularly those with CIS, pembrolizumab—which has demonstrated an approximately 40% durable complete response rate lasting ≥12 months—has been approved for clinical use [18]. Despite continued advances in therapy, more than 40% of NMIBC patients relapse within two years, and approximately 10% progress to MIBC [19].

### 1.3. Photodynamic Therapy and 5-ALA Mechanism of Action

One method showing promising clinical results in the treatment of NMIBC when standard therapies fail is photodynamic therapy (PDT) [20]. Table 1 below provides a concise comparison of treatment methods and their efficacy, along with potential adverse events.

PDT is a form of treatment based on the interaction between a photosensitizer, light, and oxygen. It involves the local or systemic administration of a photosensitive compound—a photosensitizer—that preferentially accumulates in affected tissues. The photodynamic reaction begins when the photosensitizer in the target tissue absorbs light, triggering a cascade of photochemical reactions that lead to the generation of reactive oxygen species (ROS). These highly reactive molecules cause localized tissue damage, including cancer cell death, while minimizing the impact on surrounding healthy tissues [30]. To date, PDT has been clinically approved for the treatment of various malignancies [31]. One of the key factors in PDT is the selection of an appropriate photosensitizer [32].

In photodynamic therapy using 5-aminolevulinic acid (5-ALA), the photosensitizer is the endogenously produced compound protoporphyrin IX (PpIX), formed through the administration of 5-ALA and its subsequent transformation within the heme biosynthesis pathway. 5-ALA is metabolized in cells via this intrinsic pathway, leading to the accumulation of fluorescent PpIX, which, upon light activation, generates ROS and induces cell death [33]. In physiological conditions, 5-ALA is synthesized from succinyl-CoA and glycine in a reaction catalyzed by ALA synthase, followed by a series of enzymatic steps that lead to the formation of PpIX and ultimately heme. This pathway is tightly regulated by iron availability and heme concentration.

**Figure 1:**
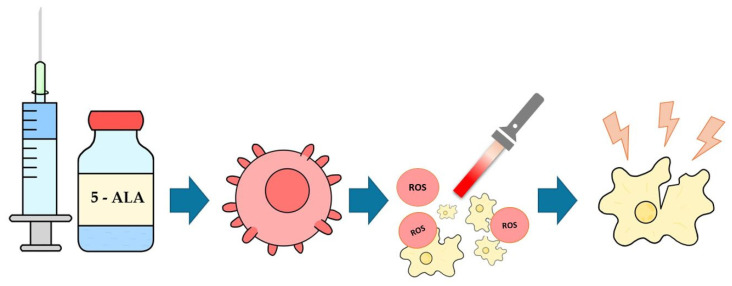
Presents mechanism of action of 5-ALA. At the beginning there is administration of 5-ALA, then accumulation of PpIX in the tumor, next ROS are generated, and, in the end, there is death of carcinoma cell.

After exogenous administration of 5-ALA (for example, in the context of PDT), PpIX accumulation within cells may result from increased substrate levels, enhanced ALA synthase activity, or dysfunction of ferrochelatase (FECH)—the enzyme responsible for catalyzing the conversion of PpIX to heme [34]. In cancer tissues, impaired FECH activity promotes the accumulation of PpIX and explains its preferential retention in tumor cells. Reduced FECH expression has been observed in colorectal cancer liver metastases, prostate cancer, and both bladder and colorectal carcinomas [35,36,37]. Porphobilinogen deaminase (PBGD), which catalyzes one of the key steps in porphyrin biosynthesis, also plays a crucial role in PpIX formation. Increased PBGD activity, as well as elevated activity of enzymes such as ALA dehydratase and uroporphyrinogen decarboxylase, has been reported in breast cancer, squamous cell carcinoma, and adenocarcinoma cells, correlating with enhanced PpIX accumulation [38,39,40,41,42]. The mechanism of action of 5-ALA is shown in Figure 1. PDT products, i.e., the formation of ROS and biochemical and structural changes in tissue (e.g., necrosis, edema, changes in oxygenation), affect local magnetic fields and the water proton microenvironment. Increased tissue oxygenation and changes in water–macromolecule interactions can lead to a shortening of T1 [43], while structural damage, edema, or necrosis can affect T2 relaxation by modifying local magnetic field heterogeneity and proton mobility.

### 1.4. Magnetic Resonance Imaging a Tool for Therapeutic Response

MRI enables the non-invasive detection of structural and functional differences between healthy and diseased tissues, allowing for the monitoring of treatment effects, localization of tumor lesions, and precise evaluation of tissue responses over time [44,45,46]. In the context of PDT, MRI offers the potential to quantify tissue alterations induced by the generation of ROS and subsequent cellular damage, which may not be immediately apparent on conventional imaging or cystoscopy. Previous studies, including our own, have demonstrated that the determination of spin–lattice (T1) and spin–spin (T2) relaxation times allows differentiation between healthy and cancerous tissues, as well as monitoring of PDT-induced changes ex vivo [47,48].

By measuring T1 and T2 relaxation times before and after 5-ALA-based PDT in NMIBC samples, we aimed to evaluate whether these parameters can serve as sensitive, non-invasive biomarkers of therapeutic response. Establishing such quantitative MRI markers could improve the real-time assessment of treatment efficacy, support personalized therapy planning, and potentially reduce reliance on invasive procedures. In this study, we assessed the utility of T1 and T2 relaxation time measurements using MRI for differentiating between healthy bladder tissues and NMIBC tissues before and after 5-ALA PDT under ex vivo conditions, as potential quantitative markers of therapeutic response.

## 2. Materials and Methods

### 2.1. Tissue Collection of NMIBC and Healthy Bladder

Bladder cancer tissue samples were collected during 2019–2021 at Provincial Clinical Hospital No. 1 in Rzeszów. 25 patients were between 50 and 80 years of age. Men predominated. All patients enrolled in the study had early-stage disease. TURBT is the standard treatment for NMIBC. European Association of Urology (EAU) guidelines recommend resection of bladder tumors with a clear margin of normal-appearing tissue, with consensus suggesting a circumferential margin of at least 5 mm from the tumor margin. Therefore, the healthy tissues in our project were fragments collected from the same patients as the tissues characterized as NMIBC, but they came from the margin of the resected tumor fragment. Immediately after removal, bladder cancer tissues were evaluated at the Department of Pathomorphology of the Clinical Regional Hospital No. 1 in Rzeszow. The total number of samples collected from 25 patients was 50 (25 healthy and 25 cancerous). Secured tissue sections for clinical testing were frozen in a cryostat at a temperature below −17 °C, and then transported to the tissue bank of the University of Rzeszow for storage at −72 °C. On the day of the experiment, the tissues were thawed to room temperature. In all cases, we found no expanded extracellular spaces or shrunken cells resulting from the Freeze–Thaw cycle. The study was approved by the Ethics Committee of the University of Rzeszow (protocol code 29/05/2019 and date of approval: 9 May 2019).

### 2.2. Sample Preparation

A total of 50 bladder tissue samples were used in this study. Group I (control group, n = 25) consisted of healthy bladder tissues that were neither incubated with 5-ALA (Sigma Aldrich, Warsaw, Poland) nor exposed to PDT. Group II (control group—NMIBC, n = 25) consisted of NMIBC tissues that were neither incubated with 5-ALA nor exposed to light. Group III consisted of the same tissues from group II (NMIBC_PDT, n = 25, but exposed to PDT). They were incubated in 5-ALA solution (concentration 0.003 mM) in the dark at room temperature for 180 min and then exposed to laser light irradiation.

### 2.3. Photosensitizer

In the experiment, the photosensitizer used was 5-ALA. The choice of 5-ALA was based on its favorable pharmacokinetic properties and selective accumulation in tumor cells. Unlike other porphyrin-based photosensitizers, 5-ALA is a natural precursor of protoporphyrin IX, which allows for more controlled and transient photosensitizer production within target tissues. This leads to lower systemic toxicity and faster clearance from the body, which are advantageous for clinical translation and patient safety. Additionally, 5-ALA-induced PpIX exhibits strong fluorescence, enabling both PDT and fluorescence-guided diagnosis. Given that 5-ALA is administered for the treatment of skin lesions in PDT, we wanted to test its use in NMIBC tissues and see if clinical MRI could be a useful tool in the verification and assessment of tissues after PDT with 5-ALA. The experiment used 5-ALA at a concentration of 0.003 mM treated water in an AquaB Duo reverse osmosis system (Fresenius Medical Care, Singapore) was used to prepare the photosensitizer stock solutions. Before application to NMIBC tissue samples, the solution was saturated with oxygen (99%, STP; DIN Chemicals, Bielsko-Biala, Poland) for 10 min. The low therapeutic concentration used in our study to achieve selective photosensitizer accumulation in target cells was a deliberate and intentional approach. Due to the experimental design approach, we wanted to determine whether changes in T1 and T2 relaxation times would occur even at such a low concentration. We wanted to determine whether clinical MRI could detect changes in T1 and T2 relaxation times in tissues treated with PDT and treated with 5-ALA at a concentration not used in standard research protocols. These changes were observed because the times differed before and after treatment. In our work, we wanted to test and apply unconventional approaches, which is why this low concentration was chosen.

### 2.4. Photodynamic Protocol

NMIBC tissue samples (n = 25) were individually warmed to room temperature and placed in the center of a plastic Petri dish for the addition of 5-ALA stock solution. Immediately after oxygenation, a volume of 0.1 mL of the stock solution at a concentration of 0.003 mM was topically spread on the tissue drop by drop, allowing the solution to cover the entire surface of the tissue sample. The 5-ALA-coated tissues were then covered and kept in the dark for 180 min before exposure. After this time, the 25 samples were subjected to laser irradiation (665 nm, 350 mW, Laser Power Supply model no.: PSU-III-LED, High-tech Zone, Changchun, China) with a wavelength of λ = 665 nm at 350 mW for 10 min. The distance of the light source from the surface of the tissue was selected so that the irradiation area was 2.5 × 2.5 cm^2^ and the irradiation of the samples did not cause heating of the tissue above 30 °C, as measured with a CPR-411 temperature probe (Elmetron, Zabrze, Poland). The calculated optical density is 56 mW/cm^2^. However, the calculated energy density is 33.6 J/cm^2^. The laser wavelength for 5-ALA is typically around 630 nm (red light), but can be wider, from 600 nm to 660 nm. In this laboratory experiment, a 665 nm laser with a power of 350 mW was used. Both the power and the calculated power and energy density are within the ranges of other data already published in the literature [49,50].

### 2.5. Preparation for MRI

Immediately after individual thawing, each sample was placed in a separate tube for MRI.

### 2.6. MRI Analysis of NMIBC and Bladder Tissue Samples

Longitudinal (T1) and transverse (T2) relaxation times were measured using a 1.5 Tesla Optima MR360 magnetic resonance imaging system (General Electric Healthcare, Milwaukee, WI, USA). The system is based on a superconducting magnet with a magnetic field strength of 1.5 T. The gradient system parameters, including the maximum amplitude and gradient slew rate, were 33 mT/m and 120 T/m/s, respectively. The device used software version SV23 (General Electric Healthcare, Milwaukee, WI, USA). Prepared tissue samples were scanned using a Fast Spin-Echo (FSE) sequence in the axial plane with a small flex coil. Based on the obtained DICOM images, regions of interest (ROIs) were manually delineated for each sample, and T1 and T2 relaxation times were subsequently determined. The technical parameters used in the MRI studies were as follows: scanning matrix = 320 × 224, slice thickness = 2 mm, interslice spacing = 0.5 mm, and NEX = 2. For T1 relaxation time measurements, 12 acquisitions were performed with repetition times (TR) ranging from 50 to 15,000 ms (50, 100, 200, 500, 700, 1000, 1500, 2000, 3000, 5000, 10,000, 15,000 ms) and a constant echo time (TE) of 3 ms. For T2 relaxation time measurements, 12 acquisitions were performed using identical scanning parameters except for a fixed TR of 10,000 ms and variable TE values ranging from 10 to 260 ms (10, 20, 42, 68, 85, 102, 130, 160, 200, 230, 260 ms).

**Figure 2:**
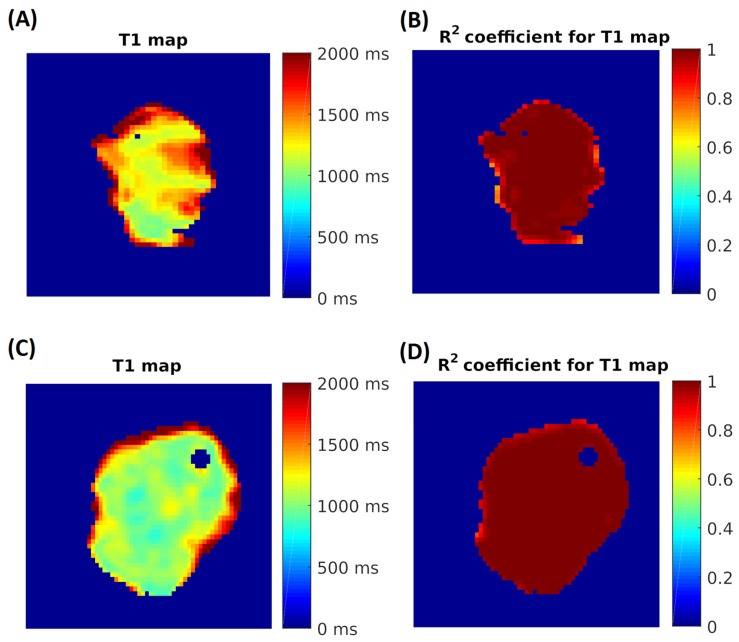
Selection of the area of interest for measured samples: (**A**) distribution of T1 longitudinal relaxation time values in NMIBC before 5-ALA-PDT; (**B**) distribution of R2 fit coefficient values; (**C**) distribution of T1 longitudinal relaxation time values in NMIBC after 5-ALA-PDT; (**D**) distribution of R2 fit coefficient values.

T1 and T2 maps (Figure 2) were generated using MATLAB software 9.14 (The MathWorks, Natick, MA, USA) for the manually segmented ROIs, employing a pixel-by-pixel three-parameter fitting algorithm via a custom interface. Color-coded T1 and T2 maps were calculated, and the mean relaxation time within each ROI was taken as the representative T1 or T2 index value.

### 2.7. Statistical Analysis

MRI data are given as mean ± SD. Statistical significance was defined as *p* = 0.05. Correlations of MRI with biochemical data were assessed using Statistica 7.0 (Ucla, Medford, MA, USA).

## 3. Results

### 3.1. Measurement of T1 Relaxation Times

**Figure 3:**
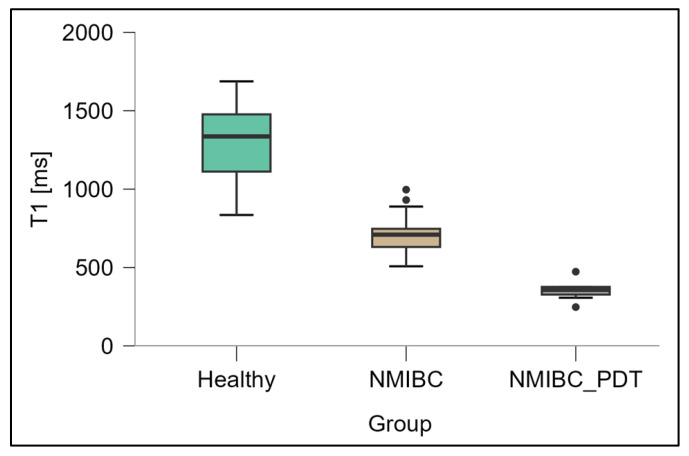
Distribution of T1 relaxation times [ms] in healthy bladder tissues (Healthy), NMIBC tissues (NMIBC), and NMIBC after PDT (NMIBC_PDT). The graph was prepared in JASP software (0.19.3.0) (University of Amsterdam, Amsterdam, The Netherlands).

The box plot in Figure 3 shows the T1 values measured for healthy bladder tissue, NMIBC tissue and NMIBC tissue after 5-ALA-PDT. The mean T1 time in the healthy tissue was 1351.7 ± 271.1 ms, while a significantly shorter relaxation time (727.7 ± 145.0 ms) was recorded in the NMIBC group, and further reduced to 368.9 ± 65.2 ms after 5-ALA-PDT. Analysis of variance ANOVA showed very strong significance of differences between groups (F = 184.4; *p* < 0.0001), and post hoc tests confirmed that all comparisons (healthy vs. NMIBC, healthy vs. NMIBC_PDT, NMIBC vs. NMIBC_PDT) were statistically significant at the *p* < 0.001 level.

### 3.2. Measurements of T2 Relaxation Times

**Figure 4:**
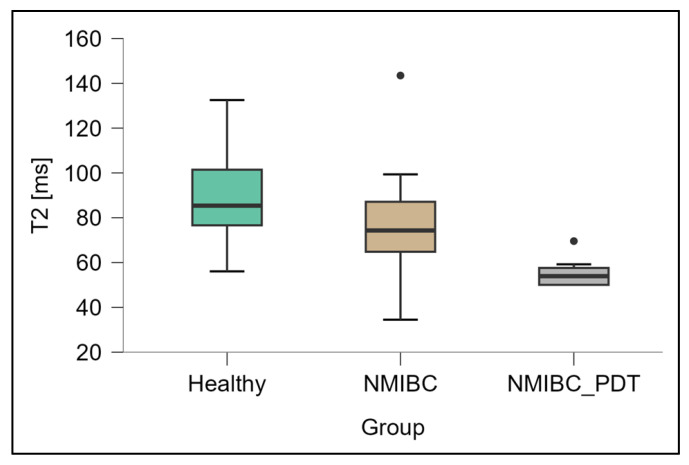
Distribution of T2 relaxation times [ms] in healthy bladder tissues (Healthy), NMIBC tissues (NMIBC) and NMIBC after PDT (NMIBC_PDT). The graph was prepared in JASP software (0.19.3.0).

The box plot in Figure 4 shows the T2 values measured for healthy bladder tissue, NMIBC tissue and NMIBC tissue after 5-ALA-PDT. The mean T2 relaxation time in the healthy tissue was 93.5 ± 20.3 ms, while the NMIBC samples recorded a value of 78.5 ± 20.4 ms, which was not significantly different. In the NMIBC_PDT group, however, the mean T2 time decreased significantly to 55.7 ± 6.1 ms. Post hoc tests showed statistically significant differences between the NMIBC_PDT group and both the healthy tissue (*p* < 0.001) and NMIBC groups (*p* < 0.01), and ANOVA analysis of variance confirmed the overall significance of the observed changes (F = 16.7; *p* < 0.0001).

A consolidated summary of relaxation-time distribution results by tissue is presented in Table 2.

## 4. Discussion

In our study, we demonstrated that measurements of T1 and T2 relaxation times are sensitive indicators of structural and biochemical changes occurring in bladder tissue under the influence of 5-ALA-PDT. First, T1 time showed a staged, statistically significant decrease: from a value of 1351.7 ± 271.1 ms in healthy tissue, through 727.7 ± 145.0 ms in NMIBC, to 368.9 ± 65.2 ms in NMIBC after 5-ALA-PDT (ANOVA: F = 184.4; *p* < 0.0001; *p* < 0.001 for all post hoc comparisons). Second, although the initial differences in T2 between healthy tissue (93.5 ± 20.3 ms) and NMIBC (78.5 ± 20.4 ms) did not reach significance, 5-ALA-PDT resulted in a marked shortening of T2 to 55.7 ± 6.1 ms (ANOVA: F = 16.7; *p* < 0.0001; *p* < 0.001 vs. healthy, *p* < 0.01 vs. NMIBC). These results clearly confirm that both T1 and T2 can serve as non-invasive markers for monitoring the efficacy of in situ PDT.

Comparison of our results with the existing literature faces significant limitations, primarily related to the sparse data on measurements of T1 and T2 relaxation times in healthy bladder tissue and NMIBC. In our ex vivo study, the measured T1 time for healthy bladder tissue (1351.7 ± 271.1 ms) significantly exceeds the value reported in vivo by Yalcin et al. (989.40 ± 81.13 ms), who, however, studied only the bladder wall [51]. These differences are multifactorial: first, ex vivo measurements are performed at room temperature, which decreases the mobility of water molecules and prolongs the longitudinal relaxation time of T1 [52]; second, under in vivo conditions, dynamic blood perfusion—through continuous exchange of water between vessels and tissue—promotes T1 shortening [53]; finally, tissue dehydration after excision may further prolong T1 in ex vivo studies [54]. To date, neither T2 measurements for bladder tissue nor detailed T1 and T2 values in NMIBC have been published. In an in vivo study by Cai et al. on urothelial carcinoma without differentiation of stage, it was observed that T1 was 1425 ± 145 ms for high-malignant lesions and 1593 ± 222 ms for low-malignant lesions, while T2 values were 106 ± 19 ms and 124 ± 23 ms, respectively [55]. All of these times were higher than in our ex vivo model. As mentioned above, the healthy bladder tissue we studied ex vivo showed longer relaxation times than the in vivo measurements, which is in line with general theories regarding the effects of temperature and perfusion on relaxation, while we observed an inverse relationship for NMIBC, which highlights the complexity and difficulty of interpreting the results obtained. To the best of our knowledge, T1 and T2 measurements in NMIBC tissues after PDT have not yet been published. Previous studies by our team have shown that PDT causes a significant decrease in both T1 and T2 in prostate cancer tissues [47], which partially correlates with the significant decrease in T1 we observed after 5-ALA-PDT of NMIBC. Our results demonstrate significant changes in T1 and T2 relaxation times after 5-ALA-PDT application in NMIBC tissue ex vivo, reflecting structural and biochemical changes in the tissue. These results confirm that PDT relies on ROS generation in tumor tissue. However, hypoxic areas in bladder tumors may limit the efficacy of conventional PDT. Recent studies on organic semiconductor photosensitizers indicate that type I PDT mechanisms can overcome such hypoxia, generating ROS even under low-oxygen conditions [56]. This suggests that integrating hypoxia-tolerant photosensitizers or optimizing PDT protocols could further improve therapeutic outcomes [56]. It is also worth noting the recent advances in nanoscale therapeutic platforms combining photodynamic and photothermal modalities, proposed by Lv et al. Although their work focuses on antibacterial applications, the mechanistic insights are relevant to oncological PDT. The proposed system enhances ROS generation and tissue heating, thus overcoming hypoxia-induced resistance and improving light–tissue interaction. Translating this concept to NMIBC therapy could enhance the extent of structural/tissue changes detected by MRI after PDT. In our study, the significant shortening of T1 and T2 times observed may reflect ROS-mediated cell death. The use of nanoscale platforms could enhance the therapeutic response of PDT already detected by MRI [57]. Moreover, recent advances in nano-enzyme-based platforms indicate that imaging and monitoring of therapeutic response can be significantly improved with materials that respond to the tumor microenvironment. Wang et al. describe nano-enzymes designed for multimodal imaging—responsive to hypoxia, acidic pH, and elevated ROS concentrations in tumors—that enable enhanced image-guided therapy. These findings are particularly relevant for NMIBC treated with 5-ALA-PDT, where difficulty in tumor oxygenation represents a barrier to efficient ROS production and, therefore, therapeutic efficacy. Integration of such strategies could also increase sensitivity when determining changes in T1 and T2 relaxation times to detect subtle tissue changes after PDT, for example, in future in vivo clinical trials [58].

### 4.1. Validation of Ex Vivo Finding in Clinical Settings

Evaluating changes in T1 and T2 relaxation times allows for the assessment of tissue composition, pathology, and response to treatment. This technique is rarely performed in clinical trials, as radiologists are primarily interested in visually examining the anatomical structures of organs. However, in the context of our experiment, we wanted to go a step further and present the diagnosis of NMIBC based on an unconventional application of MRI: the evaluation of changes in T1 and T2 relaxation times. T1/T2 changes reflect important biological processes, and the implemented PDT induces structural and cellular changes (edema, necrosis, cell death), which manifest as measurable changes in relaxation times—potentially serving as biomarkers of therapeutic effect. MRI of the urinary bladder is clinically available and performed, so monitoring the effects of 5-ALA PDT noninvasively, instead of relying on biopsies, is conceptually feasible. Implementing the presented approach in vivo requires considering certain parameters that are not present in vitro. The first aspect is artifacts caused by patient movement and natural physiological ones. Patient movement, breathing, and bladder filling cause distortions of quantitative maps, which reduces measurement accuracy. Additionally, they can affect the reliability of the measured T1 and T2 relaxation times. Another aspect is spatial resolution and tissue heterogeneity. Lesions in NMIBC are small and superficial, and the bladder wall is thin—achieving sufficient resolution without partial volume effects is difficult. Another aspect is temperature dependence [59]. Conducting ex vivo studies at room temperature may yield different results than in vivo studies. Perfusion is another factor in in vivo studies. In vivo tissues are oxygenated and undergo dynamic blood flow and exchange. These factors influence tissue composition, water mobility, microstructure, and oxygenation status, all of which influence relaxation time (through changes in molecular motion, exchange rates, and susceptibility effects). Tissue ex vivo at room temperature lacks these dynamic characteristics, so relaxation values may not reflect the in vivo microenvironment. Signal-to-noise ratio and scan time. Quantitative T1 and T2 mapping requires multiple acquisitions [60], which increases scanning time and reduces data quality in the clinical setting. In ex vivo, tissues (under appropriate conditions) can be measured several times, testing different sequences and acquisitions. In in vivo, time is not an ally, as multi-hour examinations can adversely affect patient performance. Standardization and reproducibility are also lacking. Relaxation times depend on sequence parameters, field strength, and scanner conditions. Currently, there are no standardized protocols or reference values that allow for consistent comparisons between centers. Each examination approach is different, and the most precise sequence parameters are constantly sought. It’s worth noting that T1 and T2 changes are nonspecific—they can result from necrosis, edema, inflammation, or fibrosis, which complicates biological interpretation after PDT. For the capabilities of MRI to become clinically useful, robust validation in clinical trials, reproducibility, and cost-effectiveness are essential. To enhance the clinical relevance and translational impact of the presented experiment, several strategies can be implemented to address these limitations. The first is a pilot in vivo validation study in an animal model subjected to 5-ALA-PDT. This study could measure T1 and T2 relaxation times before, during, and after PDT and correlate them with treatment response or histological outcome. This would allow us to assess how much in vivo values differ from ex vivo baseline values and whether the observed trends (T1/T2 shortening) persist in vivo. The second strategy involves temperature and perfusion correction factors. We suggest introducing correction or normalization factors to account for the known effects of temperature and perfusion. This approach would allow for modeling the ΔT between room and body temperature and adjust the ex vivo values accordingly. Additionally, imaging of perfusion fractions (e.g., dynamic contrast or arterial spin labeling) can normalize relaxation times to perfusion conditions, thereby reducing variability. Another approach is to harmonize and standardize the sequence and protocol. To ensure meaningful translation, imaging protocols for in vivo applications should closely reflect the conditions used ex vivo (e.g., echo times, inversion times, voxel size).

#### Future Challenges

The observed changes in relaxation time ex vivo are promising but translating them to in vivo conditions requires resolving issues related to motion, resolution, and biological interpretation. It remains to be determined whether the measured T1 and T2 changes directly correlate with treatment outcomes (tumor destruction, risk of recurrence) rather than with nonspecific inflammatory responses. Clinical trials with standardized acquisition protocols are needed to assess whether quantitative MRI can serve as a reliable tool for assessing PDT efficacy. In summary, this noninvasive technique has the potential to be implemented in vivo, but it faces numerous technical, biological, and clinical barriers. Only after these limitations are addressed and the observed relaxation time changes are confirmed as true indicators of treatment response can this technique become a practical diagnostic tool for monitoring the effectiveness of PDT in patients with NMIBC.

### 4.2. Integration of Relaxometry with Broader Imaging

One recommendation for the future is a prospective study combining T1 and T2 relaxation time determination with diffusion-weighted imaging (DWI), dynamic contrast-enhanced perfusion imaging (DCE), and molecular profiling (e.g., 5-ALA-induced PpIX fluorescence intensity, transcriptomic markers of oxidative stress, or hypoxia-related gene signatures). Such multimodal protocols could enable correlation of relaxation time changes with PDT-induced microstructural and molecular changes. This would enable identification of PDT efficacy using MRI. Another idea for future implementation is automated and quantitative region of interest (ROI) segmentation. To reduce operator error and improve reproducibility, the use of automated or semi-automated ROI segmentation algorithms based on machine learning or deep neural networks could be a viable solution [61]. Integration of such tools would facilitate the measurement procedure at the voxel level.

A promising, yet not-so-distant, idea seems to be multimodal imaging algorithms, i.e., protocols combining data from T1 and T2 relaxation times, DWI, perfusion, and molecular markers using multiparametric models based on artificial intelligence (e.g., multiparametric feature extraction and predictive modeling) [62]. Such an approach would enable the combination of imaging findings with histopathological and molecular findings. Applications of such a pilot approach in PDT monitoring could be expanded from bladder cancer to other types of cancer.

## 5. Conclusions

In this study, we demonstrated that T1 and T2 relaxation time measurements in NMIBC and NMIBC tissue following 5-ALA-PDT can be an indicator of therapeutic response after PDT. The significant shortening of T1 and T2 values in the tumor lesion observed after treatment confirms the potential of MRI relaxometry as a noninvasive biomarker of PDT efficacy. Due to the relaxometric differences between healthy and tumor tissue, identifying potential changes allows for precise planning of further interventions. Limitations of the study, such as the ex vivo model and manually segmented samples, indicate the need for further in vivo studies involving larger cohorts and automated image analysis. We hope that MRI can ultimately become an integral tool in everyday urological practice, helping to optimize the treatment of patients with NMIBC.

## Figures and Tables

**Table 1:** Comparison of treatment methods and their efficacy.

Treatment Method	Treatment Efficacy	Adverse Events	References
TURBT (alone, without intravesical therapy)	After TURBT alone, NMIBC recurrence is estimated in 48–70% of patients; the risk of progression may reach up to 17% at 1 year and up to 45% within 5 years.	The most common complications include bleeding, 2.8%, and bladder perforation, 1.3%; overall morbidity is estimated at 14% in large series.	[21,22,23]
TURBT+single, immediate intravesical chemotherapy	Meta-analysis of 7 RCTs (n = 1476) showed a 39% reduction in recurrence risk vs. TURBT alone (OR 0.61, *p* < 0.0001).	The most frequent adverse events were irritative symptoms such as dysuria in 17% and lower urinary tract symptoms (LUTS) in 7%, which required treatment; the remaining adverse events were transient and mild.	[24,25]
TURBT + BCG	IPD meta-analysis of 9 RCTs: with maintenance BCG—32% lower risk of recurrence vs. mitomycin; without maintenance, BCG performs worse (↑28% risk of recurrence).	Common adverse events include cystitis and urinary frequency; systemic effects are rare.	[26,27]
Immunotherapy (pembrolizumab, for BCG-unresponsive CIS ± Ta/T1)	In KEYNOTE-057 (Cohort A): CR 41% at 3 months; 46% of responses last ≥12 months. In Cohort B (without CIS): 12-month DFS 43.5%.	Adverse events include immune-related reactions; Grade 3–4: 13%. No deaths were reported as directly attributable to therapy in the study.	[28]
Photodynamic therapy (PDT)	Meta-analysis of 28 studies: CR 68% in unresectable NMIBC; RFS at 12 months: 71%, at 24 months: 38%. After resection: RFS at 12 months: 81%, at 24 months: 56%. Also effective after BCG failure (1-year RFS: 68%, 2-year: 56%).	Severe events were rare; the most common were photosensitivity and LUTS/bladder irritation.	[29]

**Table 2:** Tissue-dependent relaxation times.

Sample Group	T1 (ms), Mean ± SD	T2 (ms), Mean ± SD
Healthy tissue	1351.7 ± 271.1	93.5 ± 20.3
NMIBC	727.7 ± 145.0	78.5 ± 20.4
NMIBC after PDT	368.9 ± 65.2	55.7 ± 6.1

## Data Availability

The data presented in this study are available on request from the corresponding author. The data are not publicly available due to ethical issues.
